# In Vitro Anticancer Properties of Copper Metallodendrimers

**DOI:** 10.3390/biom9040155

**Published:** 2019-04-18

**Authors:** Marcin Hołota, Jakub Magiera, Sylwia Michlewska, Małgorzata Kubczak, Natalia Sanz del Olmo, Sandra García-Gallego, Paula Ortega, Francisco Javier de la Mata, Maksim Ionov, Maria Bryszewska

**Affiliations:** 1 Department of General Biophysics, Faculty of Biology and Environmental Protection, University of Lodz, Pomorska 141/143, 90-236 Lodz, Poland; marcin.holota@op.pl (M.H.); jakubmagiera92@gmail.com (J.M.); sylwia.michlewska@biol.uni.lodz.pl (S.M.); malgorzata.kubczak@biol.uni.lodz.pl (M.K.); maria.bryszewska@biol.uni.lodz.pl (M.B.); 2 Laboratory of Microscopic Imaging and Specialized Biological Techniques, Faculty of Biology and Environmental Protection, University of Lodz, Banacha 12/16, 90-237 Lodz, Poland; 3 Departamento Química Orgánica y Química Inorganica, Universidad de Alcalá, Instituto de Investigación Química “Andrés M. del Río” (IQAR), UAH, 28871 Alcalá de Henares, Spain; n.sanzdelolmo@gmail.com (N.S.d.O.); sandra.garciagallego@uah.es (S.G.-G.); paula.ortega@uah.es (P.O.); javier.delamata@uah.es (F.J.d.l.M.); 4 Instituto Ramón y Cajal de Investigación Sanitaria, IRYCIS, 28034 Madrid, Spain; 5 Networking Research Center on Bioengineering, Biomaterials and Nanomedicine (CIBER-BBN), 50015 Zaragoza, Spain

**Keywords:** copper metallodendrimers, anticancer therapeutic agent, nanocarrier, structure, cytotoxicity

## Abstract

Newly synthesized carbosilane copper dendrimers (CCD) with chloride and nitrate surface groups seem to be good candidates to be used as gene and drug carriers in anti-cancer therapy, due to their properties such as size and surface charge. Copper attached to the nanoparticles is an important element of many biological processes and recently their anti-cancer properties have been widely examined. Zeta size and potential, transmission electron microscopy (TEM), circular dichroism (CD), analysis of haemolytic activity, and fluorescence anisotropy techniques were used to characterize copper dendrimers. Additionally, their cytotoxic properties toward normal (PBMC) and cancer (1301; HL-60) cells were examined. All tested dendrimers were more cytotoxic against cancer cells in comparison with normal cells.

## 1. Introduction

Copper is an important element involved in many essential biological processes. Copper compounds are known as anti-oxidants and they have anti-bacterial and anti-fungal activity [1]. This metal is a crucial trace element necessary for the action of several enzymes and proteins, such as cytochrome oxidase, superoxide dismutase, ascorbate oxidase, and tyrosinase. Copper takes part in energy metabolism, respiration, and DNA synthesis. Biological molecules with copper are involved in oxidation-reduction reactions, reacting directly with molecular oxygen to produce free radicals. It is known that an excess or deficiency of copper causes Wilson and Menkes diseases, respectively. Therefore, to avoid the toxic effects of copper, the mechanism of its level regulation is required. Due to the fact that elevated levels of copper were observed in many types of human tumours, the attention of researchers is focused on the activity of this metal as a therapeutic anti-cancer agent [2,3,4].

Copper compounds, similar to other anti-cancer metals such as gold, silver, and ruthenium, are known as inducers of apoptosis, especially in cancer cells [4,5,6,7]. Additionally, copper complexes with phenanthroline derivatives with various alkyl chains were shown to have not only anti-tumour activity, but also anti-metastatic and anti-angiogenic activity [8]. Therefore, copper can be considered an alternative to other metal-based drugs, particularly those with platinum compounds, which have many side effects, such as neurotoxicity, ototoxicity, emetogenesis, nephrotoxicity, fatigue, petechial, alopecia, diarrhoea, anaemia [1,9,10]. Currently, copper gluconate complex co-administered with disulfiram is subject to clinical research in therapy for refractory solid malignancies [8,11]. However, the main problem in the use of copper in anti-cancer therapy is its poor water-solubility, which can significantly reduce the bioavailability of copper-based drugs [12].

Nowadays, oncology uses the achievements of nanotechnology [13,14]. Nanotechnology is a field of research dealing with the synthesis of particles with sizes not exceeding 100 nm [15,16], such as quantum dots, carbon nanotubes, and dendrimers [17,18]. Dendrimers were synthesized for the first time in the 1970s and now are quite popular in the field of drug delivery [12,19]. These nanoparticles have unique properties, such as specific structure and a high degree of monodispersity [20,21,22]. Most of them show thermal and chemical stability and a hydrophobic character, which may contribute to their interaction with biological membranes [13]. Additionally, the presence of functional groups determines their specific properties, such as size and surface charge [12,13,23]. Moreover, attaching metal molecules to the dendrimer surface can increase their water-solubility and enhance bioavailability [13]. 

In the present study, two groups of copper carbosilane metallodendrimers were tested as candidates for use in anticancer therapy.

## 2. Materials and Methods

### 2.1. Dendrimers

**Figure 1:**
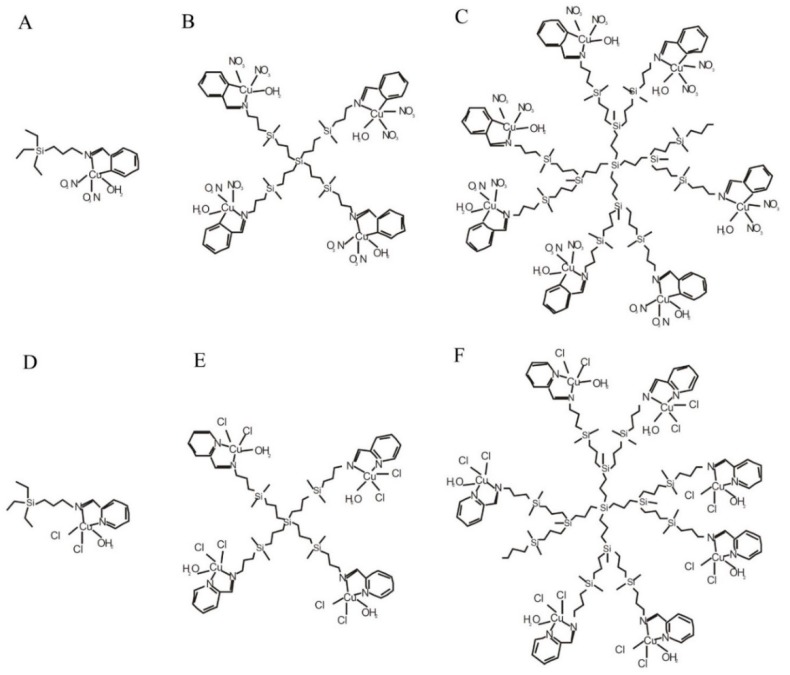
Structure of copper metallodendrimers with the nitrate (A) – CCD-NO-0, (B) – CCD-NO-1, (C) – CCD-NO-2 and chloride (D) – CCD-Cl-0, (E) – CCD-Cl-1, (F) – CCD-Cl-2- surface groups.

Two families of copper carbosilane metallodendrimers, with chloride and nitrate ligands, were used in the current study (Figure 1, Table 1).

### 2.2. Zeta Potential Technique

Zeta potential was measured using a Photon Correlation spectrometer Zetasizer Nano ZS, Malvern Instruments (UK). Helmholtz–Smoluchowski’s equation was used to calculate the data; seven measurements of five cycles of each sample were made.

### 2.3. Measurement of the Hydrodynamic Diameter of the Particles

The hydrodynamic diameter of the particles was measured using a Malvern Zetasizer Nano ZS spectrometer (UK). The dynamic light scattering technique was applied. Wavelength was set at 633 nm, a detection angle of 90°, and the refraction factor was 1.33. The measurements were conducted in distilled water. For each sample seven measurements in five cycles were made. The data were analyzed using Malvern software.

### 2.4. Transmission Electron Microscopy (TEM)

TEM was used to evaluate the structure, shape, and size of the copper metallodendrimers. Ten microliters of dendrimers at concentration 20 µmol/L were placed on 200-mesh copper grids with carbon surface. The samples were stained using uranyl acetate solution for 20 min, then washed with deionized water and dried at room temperature. The JEOL-1010 (JEOL, Akishima, Japan) transmission electron microscope was applied.

### 2.5. Circular Dichroism

Circular dichroism (CD) was assessed with the J-815 CD spectrometer (Jasco, Japan). The human serum albumin (HSA) concentration was 0.25 μmol/L. Complexes of dendrimer/HSA were prepared in a 10 mmol/L Na-phosphate buffer, pH 7.4, at molar ratios ranging from 0.5 to 10. The measurements were made from 195 to 260 nm in a Helma quartz cell with a thickness of 0.5 cm. The scan parameters were as follows: 50 nm/min scan speed, 0.5 nm step resolution, 4 s response time, 1.0 nm bandwidth, with the slit set to auto. The mean ellipticity was calculated using software provided by Jasco.

### 2.6. Haemotoxicity

Blood from healthy donors from Central Blood Bank, Lodz was used. Erythrocytes were isolated by centrifugation and washed three times with PBS 10 mM, pH 7.4. After isolation, the dendrimers in rising concentrations from 0.1 to 100 μmol/L were added to the erythrocytes with 14% hematocrit. Then the samples were incubated at 37 °C for 24 h. The absorbance was measured at 540 nm using a Jasco V-650 spectrophotometer. The percentage of haemolysis was calculated using the following formula:

H(%) = (A 540 nm/A_water_ 540 nm) × 100%.

### 2.7. Erythrocyte Membrane Isolation

To estimate the changes of membrane fluidity caused by dendrimers, the erythrocyte membranes were isolated by centrifugation (15 min, 15,000× *g*, 4 °C) and washed several times with 30 mmol/L Na-phosphate buffer, PH 7.4, diluted with water (1:1). The protein concentration was determined by the Lowry method. Final protein concentration was 0.5 mg/mL.

### 2.8. Fluorescence Anisotropy

The fluorescence anisotropy of two fluorescent probes, DPH (1,6-diphenyl-1,3,5-hexatriene) and TMA-DPH (1-[4-(trimethyl-ammonium) phenyl]-6-phenyl-1,3,5-hexatriene), intercalating in erythrocyte membranes, was measured after the addition of increasing concentrations of dendrimers using PerkinElmer LS-50B spectrofluorometer (Perkin-Elmer, Waltham, MA, USA). The excitation wavelengths were 348 nm and 358 nm and the emission wavelengths were 426 nm and 428 for DPH for TMA-DPH, respectively. The slit width of the excitation monochromator was 6 nm and that of the emission monochromator was 8 nm.

The fluorescence anisotropy values were calculated from Jablonski’s equation:

$$r=\left(I_{VV}-GI_{VH}\right)/\left(I_{VV}+GI_{VH}\right),\;$$

where *r* = fluorescence anisotropy, *I_VV_* and *I_VH_* = the vertical and horizontal fluorescence intensities, respectively, to the vertical polarization of the excitation light beam used. *G* = *I_VH_*/*I_VV_* (grating correction factor) corrects the polarization effects of the monochromator. The measurements were performed with Perkin Elmer software.

### 2.9. Cell Lines

To assay the dendrimers’ cytotoxicity, two cancer cell lines of leukaemia (HL-60 and 1301, ATCC cell lines, Manassas, Virginia, USA) and a normal cell line PMBC (peripheral blood mononuclear cells) (isolated from blood of healthy donors from Central Blood Bank, Lodz) were applied. PMBC cells were obtained from blood samples with Histopaque 1077 gradient (1500 rpm, 15 min, 24 °C) in a RPMI-1640 medium (Gibco, Thermo Fisher Scientific, Waltham, MA, USAwith 10% heat-inactivated fetal bovine serum (FBS, HyClone, GE Healthcare Life Sciences, Chicago, Illinois, USA) contained 1% of antibiotic. The cells were grown in plastic tissue culture flasks (Falcon, GE Healthcare Life Sciences, Chicago, Illinois, USA) at a temperature of 37 °C in a humidified atmosphere containing 5% CO_2_ and 95% air.

### 2.10. Cytotoxicity

To study the cytotoxicity of dendrimers at concentrations of 1–50 μmol/L, the Alamar Blue test was applied. The cells were seeded on a black 96-well plate at a density of 10,000 per well. After 24 h incubation, the absorbance/fluorescence of the samples was measured at 528 and 590 nm. Viability was estimated from the following formula:

% viability = (A/A_c_) × 100%.

### 2.11. Statistical Analysis

For the statistical analysis, the results were collected out of a minimum of three independent experiments and presented as mean ± standard deviation (SD). The Kruskal–Wallis non-parametric test was applied. Significance was accepted at * *p* < 0.05.

## 3. Results

### 3.1. Particle Size and Zeta Potential Analysis

Measurements of the dendrimers’ zeta potential provided information of their surface charges. All studied dendrimers were positively charged, and the charge values depended on dendrimer generation. The highest zeta potential was shown for the dendrimers of 2nd generation. Additionally, the zeta potential was higher for dendrimers possessing the nitrate groups than for those with chloride groups, the respective values were in the range of 14.79 ± 1.92–39.3.78 ± 3.78 mV and 10.45 ± 1.25–37.48 ± 3.09 mV (Table 2).

An analysis of the hydrodynamic diameter of dendrimers indicated that nanoparticles with chloride groups were bigger than those with nitrate groups. The respective values were in the range of 59.53 ± 8.92 to 152.13 ± 7.52 nm and 51.59 ± 6.74 to 135.28 ± 9.27 nm (Table 2). It has been shown that, in both cases, the size of dendrimers of generation 0 was higher than dendrimers of the 1st and 2nd generations. This effect can be explained by possible nanoparticle aggregation. The highest polydispersity index (PDI) values were registered for the dendrimers of generation 0 in both groups. An increase of the dendrimer generation led to a decrease of the PDI value. For dendrimers containing chlorides, PDI values were higher than for dendrimers with nitrate groups (Table 3).

### 3.2. Transmission Electron Microscopy (TEM)

**Figure 2:**
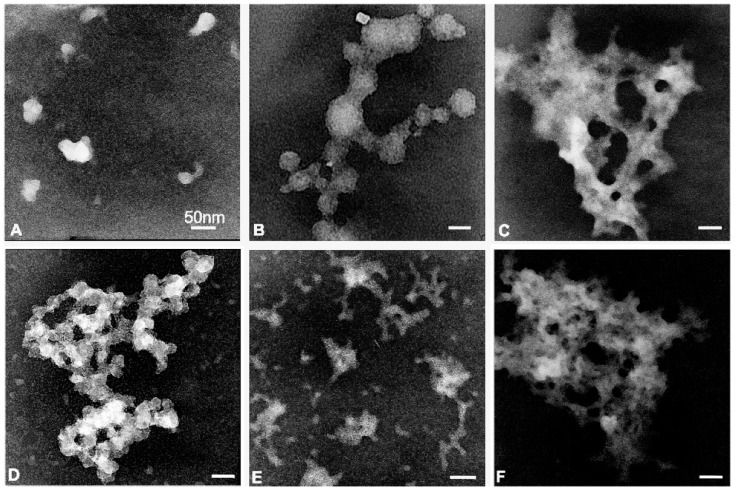
Ultrastructure of copper metallodendrimers visualized by transmission electron microscopy (TEM): (**A**) CCD-NO-0, (**B**) CCD-NO-1, (**C**) CCD-NO-2, (**D**) CCD-Cl-0, (**E**) CCD-Cl-1, (F) CCD-Cl-2. Dendrimers were dissolved in Na-phosphate buffer 10 mmol/L, pH 7.4. Bar = 50 nm.

The morphological structure of the dendrimers was analyzed using transmission electron microscopy. Opposite to the results of the Zeta technique, the smallest nanoparticles, with a size of 5–50 nm, were observed for the samples with dendrimers of generation 0 (CCD-NO-0) (Figure 2). The 1st generation dendrimer (CCD-NO-1) was visible both as a single nanoparticle of about 5–10 nm and as a bigger aggregated form. Dendrimers of the 2nd generation (CCD-NO-2) formed aggregated structures with the size of about 500 nm. In contrast, all dendrimers with chloride groups were seen as aggregated systems. Dendrimers of generation 0 (CCD-Cl-0) were visible as small, clumped structures. Dendrimers of the 1st (CCD-Cl-1) and 2nd generations (CCD-Cl-2) formed aggregate structures with a size of about 50–150 nm and 450–500 nm, respectively (Figure 2).

### 3.3. Circular Dichroism

**Figure 3:**
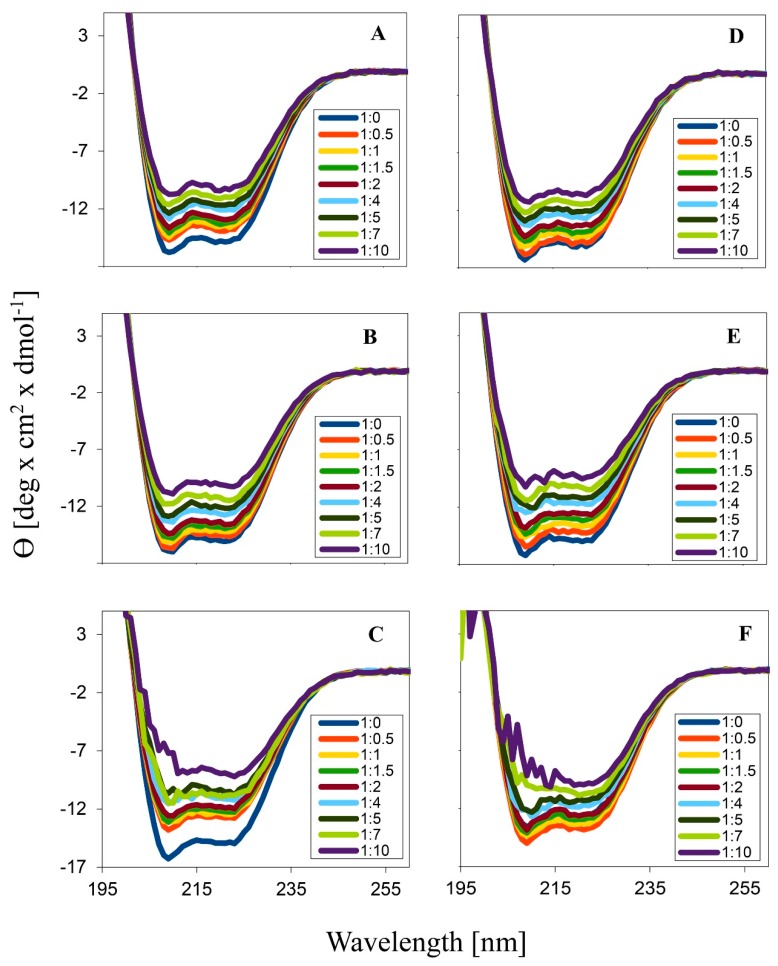
The CD spectra of human serum albumin (HSA) in the presence of copper metallodendrimers: (**A**) CCD-NO-0, (**B**) CCD-NO-1, (**C**) CCD-NO-2, (**D**) CCD-Cl-0, (**E**) CCD-Cl-1, (**F**) CCD-Cl-2. HSA concentration 0.25 μmol/L, wavelength 195–260 nm, scan speed 50 nm/min, bandwidth 1.0 nm, Na-phosphate buffer 10 mmol/L, pH 7.4.

**Figure 4:**
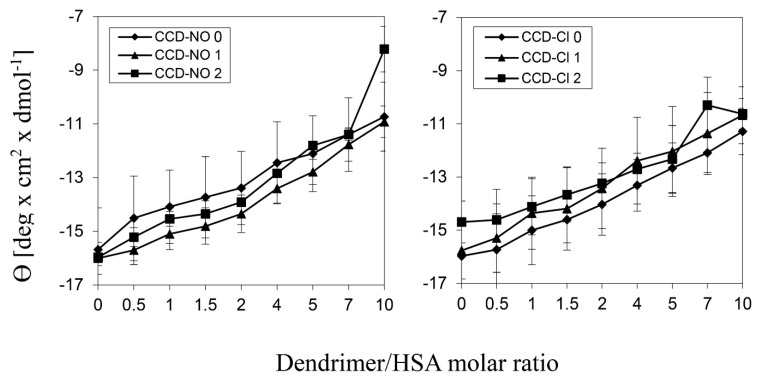
Changes in mean residue ellipticity of HSA, at λ = 210 nm in the presence of metallodendrimers. Results are mean ± standard deviation (SD), *n* = 3. HSA concentration 0.25 μmol/L, wavelength 195–260 nm, scan speed 50 nm/min, bandwidth 1.0 nm, Na-phosphate buffer 10 mmol/L, pH 7.4.

To analyze the ability of dendrimers to affect the proteins’ secondary structure we applied the circular dichroism technique. The graphics in Figure 3 indicate that the addition of increasing amounts of dendrimers into a protein solution practically did not change the typical alpha helix shape of the HSA CD spectra. Figure 4 shows the changes in mean residue ellipticity of HSA, at λ = 210 nm in the presence of CCD. The highest increase of HSA spectra ellipticity was caused by the presence of the dendrimers of the 2nd generation, for both groups. The smallest ellipticity changes were caused by the 1st generation of dendrimers with nitrate end groups and by the 0 generation of the dendrimers with chloride groups.

### 3.4. Erythrocyte Membrane Fluidity

**Figure 5:**
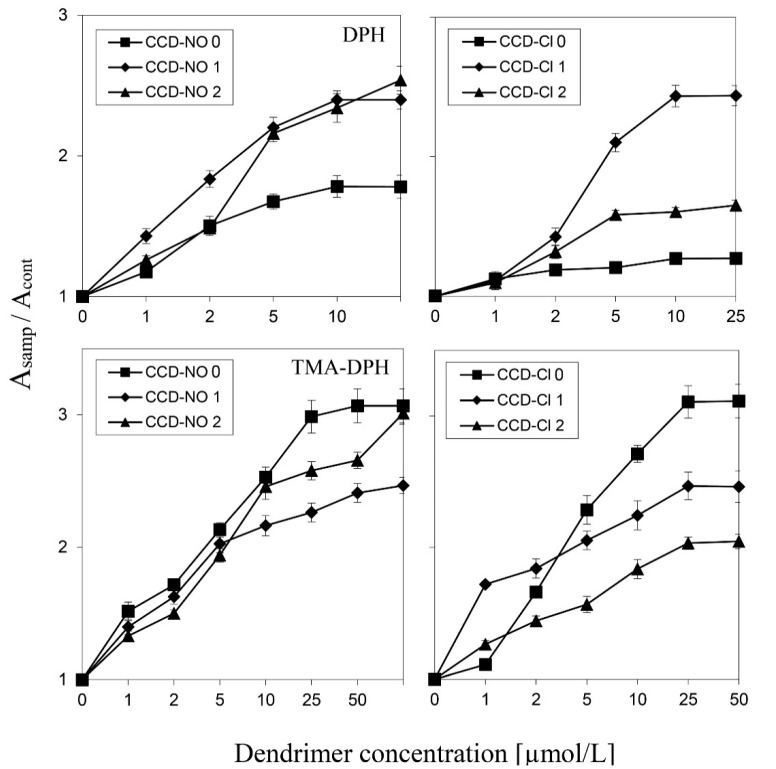
Changes in fluorescence anisotropy of DPH (top panels) and TMA-DPH (bottom panels) of erythrocyte membranes incubated with copper metallodendrimers at rising concentrations from 1 to 70 µmol/L. PBS buffer, pH 7.4, 37 °C. CCD-Cl: left panels, CCD-NO: right panels. The values are the mean ± SD, *n* = 3.

To estimate the way CCD dendrimers interact with biological membranes, the fluorescence anisotropy technique using of DPH and TMA-DPH fluorescent probes was applied. By this method, it is possible to analyze which part of the lipid membrane can be influenced by the dendrimers. The fluorescence anisotropy of the DPH probe reflects the fluidity state of the hydrophobic region of the bilayer, whereas changes in the TMA-DPH probe anisotropy show the fluidity changes in the region of the membrane surface. The results indicate that all tested dendrimers increased the fluorescence anisotropy of both probes. In the case of the dendrimers with nitrate groups, the highest anisotropy values of the DPH probe were registered after the addition of the 1st and 2nd generation dendrimers, and the lowest by the generation 0, while in the case of the TMA-DPH probe the highest increase was caused by the dendrimer of generation 0 and lowest of generation 1 (Figure 5, left panels).

Dendrimers with chloride groups caused the highest increase in DPH probe anisotropy in the case of the CCD-Cl-1 dendrimer, while for the other two the parameter was just slightly changed. TMA-DPH fluorescence anisotropy was the highest in the presence of CCD-Cl-0 and the smallest for CCD-Cl-2 dendrimers (Figure 5, right panels).

### 3.5. Hemotoxicity

**Figure 6:**
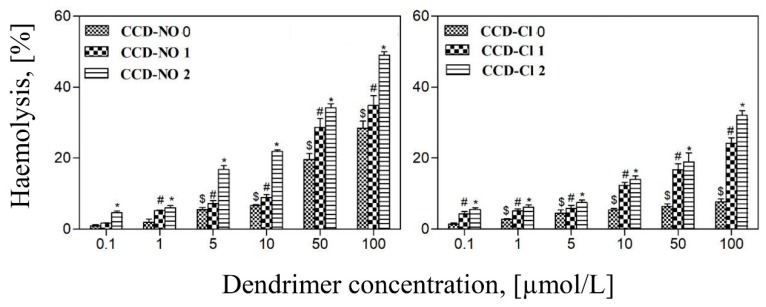
Erythrocyte haemolysis induced by copper metallodendrimers after 24 h of incubation. The concentration range 0.1–100 µmol/L. 2% haematocrit in PBS buffer, pH 7.4, 22 °C. Results are mean ± SD, *n* = 6.

The hemotoxicity test was used to study the interaction of dendrimers with the erythrocyte membrane. Destruction of the membrane triggers the release of proteins, including haemoglobin. Figure 6 presents the results of erythrocyte haemolysis caused by CCD dendrimers after 24 h incubation. The intensity of membrane destruction depended on the dendrimer kind, generation, and applied concentration. Results show that at lower concentrations dendrimers with chloride groups were more hemotoxic than those with nitrate groups. However, along with the increase of concentration the opposite result was observed, where the effect of CCD-NO dendrimers was higher.

### 3.6. Cytotoxicity

**Figure 7:**
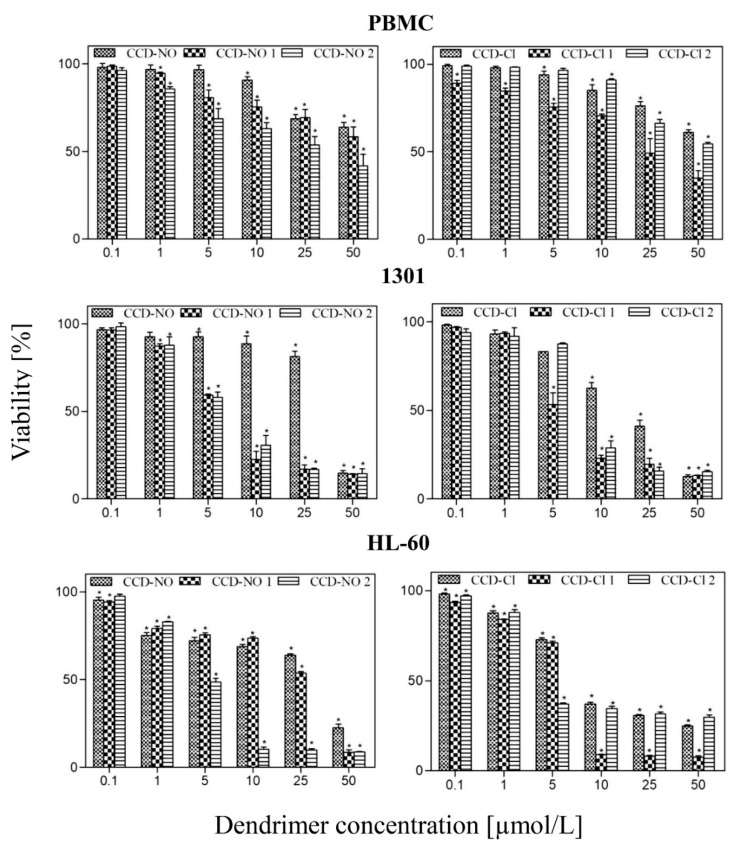
Effect of copper metallodendrimers on the viability of normal PBMC and cancer: 1301, HL-60 cell lines after 24 h incubation. The concentration range was 0.1–50 µmol/L. The values are the mean ± SD of *n* ˃ 6.

The influence of the CCD dendrimers on normal PBMC and cancer 1301 and HL-60 cells was evaluated (Figure 7). The performed cytotoxicity tests showed that the tested compounds affected the PBMC viability less than cancer cells. In contrast, they caused concentration- and generation-dependent decreases in the viability of both cancer cell lines. All dendrimers were more cytotoxic to 1301 than to HL-60 cell line. The concentration up to 1 μmol/L did not decrease the viability of the cancer cell lines. CCD-NO-1 and CCD-NO-2 dendrimers at a concentration of 5 μmol/L decreased 1301 cell viability up to 59.2% and 58.0% compared to the control, respectively. The increase in dendrimer concentration resulted in a drop of the 1301 cell viability to 14.2% and 14.6% compared to the control, respectively. Similarly, the 2nd generation dendrimer (CCD-NO-2) at concentrations 5–50 μmol/L caused a decrease in the HL-60 cell viability up to 48.4%–8.7% more than the control. The 1st generation dendrimer (CCD-NO-1) from 0.1 to 5 μmol/L did not significantly affect the HL-60 cell viability. An increase in its concentration up to 50 μmol/L decreased cell viability to 8.4% of the control. The dendrimer of generation 0 (CCD-N-0) caused a statistically significant decrease in both 1301 and HL-60 cell viability to 14.8% and 22.4% compared to the control, respectively, however only at a concentration of 50 μmol/L.

Dendrimers with chloride groups (CCD-Cl-1), at a concentration of 5 μmol/L, significantly decreased the viability of 1301 cells to 53.7% compared to the control, while the effect of generation 0 (CCD-Cl-0) and generation 2 (CCD-Cl-2) at same concentration was smaller—cell viability decreased up to 82.9% and 87.5%, respectively. At a concentration of 10 μmol/L, the dendrimers of generation 0 and 2 decreased the viability of 1301 cells up to 62.5% and 28.7%, respectively. The treatment of 1301 cells with all chloride dendrimers, CCD-Cl-0, CCD-Cl-1, and CCD-Cl-2, at a concentration of 50 μmol/L decreased their viability up to 12.6%, 13.3%, and 15.4%, respectively. The viability of HL-60 cells treated with 5 μmol/L of CCD-Cl-2 decreased up to 37.6% compared to the control, then with increasing dendrimer concentrations up to 50 μmol/L, the cells’ viability did not change significantly. CCD-Cl-0 generation 0 and CCD-Cl-1 generation 1 at concentrations of up to 50 μmol/L decreased cell viability to 25% and 7.6%, respectively. The IC_50_ values of each dendrimer for all studied cell lines considered in this study are summarized in Table 4.

## 4. Discussion

Copper is one of the anti-cancer metals and due to low toxicity seems to be an interesting alternative in cancer therapy. In this paper we investigated its biophysical properties and cytotoxicity to normal and cancer cells of carbosilane dendrimers containing copper molecules in their structure (Figure 1).

Measurements of the size of CCD dendrimers using the dynamic light scattering technique showed that dendrimers of generation 0 (CCD-NO-0) and (CCD-Cl-0) were the largest in their respective groups, while in other generations this parameter depended on generation. This effect can indicate a trend to aggregate the dendrimers of generation 0. A similar tendency was described for the carbosilane ruthenium dendrimers (CRD) of generation 0. This tendency can be due to electrostatic interactions between single molecules of dendrimers. On the contrary, TEM analysis showed that dendrimers of generation 0 were seen as single 5–50 nm nanoparticles (Figure 2.). This discrepancy between sizes of nanoparticles is probably due to the different methods applied. The zeta size measurements were conducted in a solution while TEM was conducted in a dry state [13,24].

The measurements of the zeta potential in a solution can provide information about dendrimer surface charges. The tested nanoparticles were positively charged. Similar results were described earlier in the experiments with CRD dendrimers [13]. It is known that the positive charge makes interaction with negatively charged biological membranes easier [13,25,26,27]. Due to this positive charge, cationic dendrimers were shown to be cytotoxic for normal Hippo-18 cells [28].

Analysis of the fluorescence anisotropy changes of two fluorescent labels, DPH and TMA-DPH, that are located at different membrane depths, confirmed that all tested dendrimers interacted with both regions of membranes and changed membrane fluidity. The observed increase in the fluorescence anisotropy indicated the rising membrane stiffness [29], which in turn reflected the ability of the tested dendrimers to interact with bilayers [30,31]. Phosphorothioate dendrimers of the 5th generation with peripheral hydroxyl groups [32] and viologen-phosphorus dendrimers were also shown to enhance the stiffness of biological membranes, although the intensity of this process was lower [33].

Next, to check the effect of dendrimers on the cell membrane, the degree of haemolysis was determined. Damage to the membrane caused by interaction with ligands results in the outflow of proteins, in particular haemoglobin [13]. Carbosilane copper dendrimers caused haemolysis after 24 h of incubation, the intensity of haemolytic effect depended on their generation and concentration. At lower concentrations, the dendrimers with the chloride ligands were more efficient than those with nitrate ones. However, with rising concentrations the opposite effect was observed. The lowest efficiency of the 0 generation dendrimers may have resulted from the smaller number of active groups compared to the higher generation dendrimers [13,34,35,36,37]. A similar effect was demonstrated in the experiments with a carbosilane dendrimer terminated with ruthenium [13], phosphoric dendrimers [38], and PAMAM dendrimers [35]. In contrast, after 24 h incubation, the haemolytic activity of the dendrimers containing titanium was not observed [39]. The surface charge of dendrimers is therefore an important haemotoxicity agent [38]. Cationic dendrimers can interact strongly with negatively charged membranes and can be more toxic compared to neutral or anionic dendrimers [20,34,40].

Circular dichroism spectroscopy can be used to check the interaction between dendrimers and albumin and their effect on the secondary structure of this protein [41]. CCDs caused minor changes to the albumin secondary structure. The addition of the increasing dendrimer concentrations to the HSA solution did not change the characteristic alpha-helix spectrum, while an increase in ellipticity was observed. This is advantageous because a large change in the original protein structure in the complexes would probably result in a loss of the biological activity of the protein, causing a limitation in the potential medical use [42].

The impact of copper-terminated carbosilane dendrimers on the viability of PBMC normal cells and 1301, HL60 cancer ones was evaluated and the dendrimers’ cytotoxicity was checked. It is worth noting that all dendrimers were significantly more toxic to both cancer cell lines than to PBMCs.

The cytotoxic effect depended on the kind, generation, and concentration of the dendrimers. Among all tested dendrimers the dendrimer of the 1st generation with chloride groups (CCD-Cl-1) was the most toxic to HL-60. The same dendrimer was equally toxic to the human prostate cancer cell line PC3 [1]. However, Brahmi et al. [43] showed that the toxicity of phosphorus dendrimers with copper increased with its generation. Similarly, a generation-depended impact of ruthenium-terminated carbosilane dendrimers (CRD) on HL-60 cells was demonstrated [13]. The obtained findings are in good agreement with the previous studies [44,45,46], which suggests the importance of polymeric and hybrid nanoparticles as efficient carriers in drug delivery.

## 5. Conclusions

All studied CCD dendrimers strongly interact with cell membranes. They show significantly higher toxicity to tumour cells compared to normal cells. The cytotoxicity of dendrimers is concentration- and generation-dependent. Dendrimers with a nitrate ligand are more toxic than chloride dendrimers. On the basis of the obtained results, it can be proposed that the studied dendrimers are an alternative to other non-viral carriers to be used in classic anti-cancer therapy.

## Figures and Tables

**Table 1:** Characterisation of copper metallodendrimers with chloride and nitrate surface groups.

	CCD-NO-0	CCD-NO-1	CCD-NO-2	CCD-Cl-0	CCD-Cl-1	CCD-Cl-2
Generation	0	1	2	0	1	2
Surface groups number	1	4	8	1	4	8
Molecular weight [g/Mol]	468.04	1840.10	3992.90	414.93	1627.68	3696.01
Solubility	MeOH/DMF/DMSO			DMF/DMSO/CHCl_3_/CH_2_Cl_2_		

**Table 2:** Zeta potential and zeta size of copper metallodendrimers with nitrate and chloride surface groups.

Dendrimer	Zeta Potential, [mV]	Zeta Size, [nm]
**CCD-NO-0**	14.79 ± 1.92	135.28 ± 9.27
**CCD-NO-1**	25.90 ± 2.32	51.59 ± 6.74
**CCD-NO-2**	39.23 ± 3.78	63.12 ± 5.28
**CCD-Cl-0**	10.45 ± 1.25	152.13 ± 7.52
**CCD-Cl-1**	19.68 ± 1.78	59.53 ± 8.92
**CCD-Cl-2**	37.48 ± 3.09	74.27 ± 7.26

Means ± SD.

**Table 3:** Polydispersity index (PDI) of copper metallodendrimers with nitrate and chloride surface groups.

Dendrimer	PDI
**CCD-NO-0**	0.537 ± 0.146
**CCD-NO-1**	0.370 ± 0.087
**CCD-NO-2**	0.229 ± 0.022
**CCD-Cl-0**	0.542 ± 0.122
**CCD-Cl-1**	0.423 ± 0.068
**CCD-Cl-2**	0.356 ± 0.062

Means ± SD.

**Table 4:** IC_50_ values of copper metallodendrimers in normal peripheral blood mononuclear cell (PBMC) and cancer: 1301, HL-60 cell lines after 24 h incubation. The values are the mean ± SD of *n* ˃ 6.

Dendrimer	PBMC	1301	HL60
**CCD-NO-0**	62.64 ± 0.2	27.84 ± 1.2	29.05 ± 0.6
**CCD-NO-1**	56.55 ± 0.4	5.03 ± 0.2	24.32 ± 1.7
**CCD-NO-2**	35.02 ± 0.2	4.85 ± 2.4	4.01 ± 0.4
**CCD-Cl-0**	62.23 ± 0.3	12.44 ± 0.7	5.94 ± 0.4
**CCD-Cl-1**	31.57 ± 7.3	4.58 ± 2.4	5.57 ± 0.2
**CCD-Cl-2**	50.95 ± 1.1	7.12 ± 0.3	2.37 ± 0.3

Means ± SD.
